# Inflammatory Biomarkers Predict Local Control and Survival After Escalated High-Dose SBRT in Borderline and Locally Advanced Pancreatic Cancer

**DOI:** 10.3390/jcm14186573

**Published:** 2025-09-18

**Authors:** Laura Ferrera-Alayon, Antonio Alayón Afonso, Bárbara Salas-Salas, Nereida Rodríguez-González, Pedro C. Lara, Marta Lloret-Saez-Bravo

**Affiliations:** 1 Department of Radiation Oncology, University Hospital Dr Negrín Las Palmas de Gran Canaria, Barranco de la Ballena s/n, 35010 Las Palmas de Gran Canaria, Spainnrodgon@gobiernodecanarias.org (N.R.-G.); 2 Canarian Institute for Cancer Research, 380204 San Cristobal de La Laguna, Spain; 3 Canarian Comprehensive Cancer Center, Department of Oncology, University Hospital San Roque, C. Dolores, de la Rocha, 5, 35001 Las Palmas de Gran Canaria, Spain; 4 Department of Medicine, Fernando Pessoa Canarias University, Calle la Juventud, s/n, 35450 Santa Maria de Guía, Spain; 5 Department of Clinical Medicine, Las Palmas de Gran Canaria University, Juan de Quesada, 35001 Las Palmas de Gran Canaria, Spain

**Keywords:** neoadjuvant, SBRT, escalate high dose, BRPC, LAPC

## Abstract

**Background**: Inflammatory biomarkers such as the platelet-to-lymphocyte ratio (PLR) and neutrophil-to-lymphocyte ratio (NLR) have been increasingly investigated as prognostic indicators in pancreatic cancer. However, their role in patients receiving high-dose neoadjuvant stereotactic body radiotherapy (SBRT) remains unclear. **Methods**: Thirty-three patients with borderline resectable (BRPC) or locally advanced pancreatic cancer (LAPC) prospectively included from June 2017 to December 2022 in a multicenter academic SBRT escalated-dose study of neoadjuvant chemotherapy followed by escalated-dose SBRT (50–55 Gy in 5 fractions) were scored according to PLR/NLR expression, before SBRT. Patients were stratified according to the median value for each marker. The primary endpoint was freedom from local progression as the first site of failure (FFLP-FF). Secondary endpoints included cancer-specific survival (CSS) and overall survival (OS). Follow-up was conducted prior to the closing date of 18 July 2025. **Results**: After a mean follow-up of 24 months (range 6–71 months), the two-year FFLP-FF rate for the entire cohort was 80.2%. High PLR prior to SBRT was significantly associated with lower FFLP-FF (*p* = 0.038). Similarly, elevated NLR was associated with reduced FFLP-FF (*p* = 0.014). Patients with both high PLR and high NLR showed the poorest FFLP-FF outcomes (*p* = 0.001). High pre-SBRT PLR was also correlated with reduced CSS (*p* = 0.019) and OS (*p* = 0.018). **Conclusions**: Pre-treatment inflammatory biomarkers, particularly PLR and NLR, may serve as valuable predictors of local control and survival in patients with borderline or locally advanced pancreatic cancer undergoing escalated high-dose SBRT. Their combination may help identify subgroups with a worse prognosis who may benefit from tailored treatment strategies.

## 1. Introduction

Pancreatic ductal adenocarcinoma (PDAC) is one of the most lethal malignancies worldwide, ranking as the seventh leading cause of cancer-related death and accounting for over 466,000 deaths annually [1]. Despite advances in diagnostic imaging, surgical techniques, and systemic therapies, the five-year overall survival rate for PDAC remains below 10%, reflecting the aggressive biology of the disease and the frequent diagnosis at advanced stages [1,2]. Most patients present with locally advanced or metastatic disease, and only a minority are candidates for potentially curative resection at diagnosis [2].

For patients with borderline resectable (BRPC) or locally advanced pancreatic cancer (LAPC), the possibility of achieving an R0 resection is often limited by vascular invasion or extensive local involvement [2]. However, recent evidence from the CONKO-007 randomized trial demonstrated that neoadjuvant treatment was associated with significantly improved R0 resection rates and survival outcomes in patients with resectable and borderline pancreatic cancer [3]. In this context, neoadjuvant strategies have gained increasing attention, aiming to improve surgical outcomes and long-term survival. The combination of multi-agent chemotherapy and stereotactic body radiotherapy (SBRT) has emerged as a promising approach, particularly in high-volume centers and within clinical trials [4]. SBRT allows for the delivery of high radiation doses to the tumor with minimal exposure to surrounding healthy tissues, potentially enhancing local control while reducing toxicity [4,5]. Several prospective studies and meta-analyses have demonstrated the feasibility, safety, and efficacy of dose-escalated SBRT in patients with BRPC and LAPC, reporting encouraging rates of local control, as well as tumor downstaging [4,5,6].

Our group has recently contributed to this field with a prospective multicenter study evaluating dose-escalated SBRT in BRPC and LAPC. We demonstrated that delivering higher biological effective doses (BEDs) with SBRT is feasible and safe, resulting in promising rates of local control and pathological response [1]. These results support growing evidence that dose escalation in SBRT may be a key factor in improving local tumor control and surgical outcomes in this challenging patient population.

However, optimal patient selection for these intensified neoadjuvant strategies remains a significant challenge. Traditional clinical and radiological criteria, such as tumor size, vascular involvement, and performance status, do not always capture the underlying tumor biology or the host’s systemic response to cancer [7]. As a result, there is a growing interest in identifying accessible and reliable biomarkers that can improve prognostic stratification and guide personalized treatment decisions [8,9].

Systemic inflammation has been recognized as a key factor in cancer progression, influencing tumor growth, invasion, metastasis, and response to therapy [9,10]. In this context, systemic inflammatory biomarkers such as the neutrophil-to-lymphocyte ratio (NLR) and platelet-to-lymphocyte ratio (PLR) have emerged as promising prognostic indicators in various solid tumors, including pancreatic cancer [8,9,10]. These markers, easily obtained from routine blood counts, reflect the balance between pro-tumor inflammatory processes and anti-tumor immune responses [10]. Elevated NLR and PLR have been associated with increased tumor aggressiveness, immunosuppression, and poor prognosis in both resectable and advanced pancreatic cancer [9,11].

The biological rationale for the prognostic value of NLR and PLR is supported by evidence that neutrophils can promote tumor progression through the secretion of cytokines, growth factors, and proteases, while lymphocytes play a crucial role in anti-tumor immunity [10,11]. However, neutrophils have also been described as exerting a dual or ‘Janus-like’ role in tumor biology, potentially contributing to anti-tumor immunity through cytotoxic activity and the modulation of adaptive responses [12,13]. Similarly, platelets can facilitate tumor cell survival, angiogenesis, and metastasis, further contributing to disease progression [10]. Thus, a high NLR or PLR may indicate a pro-tumor inflammatory state and a suppressed immune response, both of which are associated with worse clinical outcomes [9,10,11].

Several studies have evaluated the prognostic significance of NLR and PLR in pancreatic cancer. Meta-analyses and large cohort studies have consistently shown that high pre-treatment NLR and PLR are associated with worse overall survival, higher recurrence rates, and a reduced response to therapy [12,13]. For example, Ganbarli et al. [8] and Martin et al. [9] demonstrated that elevated NLR and PLR are independent predictors of poor prognosis in both resectable and unresectable PDAC. Furthermore, Szkandera et al. [11] validated the prognostic value of NLR in a large cohort of pancreatic cancer patients, while Yang et al. [10] and Cheng et al. [14] confirmed these findings in meta-analyses including thousands of patients.

Recent evidence also suggests that the combination of NLR and PLR may provide additional prognostic information, identifying subgroups of patients with particularly poor outcomes [15]. García-Herrera et al. [15] reported that patients with both elevated NLR and high CA 19-9 levels had significantly worse survival, highlighting the potential utility of combining inflammatory and tumor-specific biomarkers for risk stratification.

Despite the growing body of evidence supporting the prognostic value of NLR and PLR in pancreatic cancer, most studies have focused on patients undergoing surgery or systemic therapy, with limited data available for those treated with neoadjuvant SBRT [4,5,6]. The specific role of these biomarkers in predicting outcomes such as freedom from local progression as first failure (FFLP-FF), overall survival (OS), and cancer-specific survival (CSS) in the context of dose-escalated SBRT remains underexplored.

Given the increasing use of neoadjuvant SBRT in BRPC and LAPC, as well as the need for improved prognostic tools to guide clinical decision-making, further research is warranted to clarify the utility of NLR and PLR in this setting. The identification of reliable, non-invasive biomarkers could facilitate personalized treatment strategies, ultimately improving patient outcomes in relation to this challenging disease.

Therefore, the present study aims, for the first time, to evaluate the prognostic value of pre-SBRT NLR and PLR in patients with BRPC or LAPC treated with dose-escalated SBRT, analyzing their association with freedom from local progression as first failure (FFLP-FF), cancer-specific survival (CSS), and overall survival (OS). Additionally, we explore the impact of the combined elevation of these markers to refine risk stratification in this clinical scenario.

## 2. Materials and Methods

Patients with borderline resectable (BRPC) or locally advanced pancreatic cancer (LAPC) prospectively included in an academic multicenter study of neoadjuvant chemotherapy followed by escalated-dose SBRT, from June 2017 to December 2022 [1], were scored according to their PLR/NLR expression before SBRT.

### 2.1. Study Population

In short, this cohort included 33 patients with a median age of 61.7 years (range: 37–82), of whom 57.6% were female [1]. All patients had histologically confirmed pancreatic adenocarcinoma, with no evidence of distant metastasis at diagnosis. Tumors were classified as borderline resectable (BRPC) in 39.4% and locally advanced (LAPC) in 60.6% of cases. Most patients were staged as T3 (45.5%) or T4 (36.3%), and the majority were node-negative (75.8%). Tumor staging was conducted according to the 8th edition of the TNM classification system [16,17]. Baseline clinical and demographic characteristics are summarized in Table 1.

As previously described [1], patient neoadjuvant chemotherapy was administered by the Medical Oncology Departments of these university hospitals, according to the standard of care of each institution. Two standard regimens were used—(1) modified FOLFIRINOX (mFOLFIRINOX), administered every two weeks and consisting of oxaliplatin (85 mg/m^2^), irinotecan (180 mg/m^2^), leucovorin (400 mg/m^2^), and a 46 h infusion of fluorouracil (2400 mg/m^2^); (2) gemcitabine plus nab-paclitaxel (GEM-ABX), which includes gemcitabine (1000 mg/m^2^) and nab-paclitaxel (125 mg/m^2^) on days 1, 8, and 15 of a 28-day cycle. In clinical practice, mFOLFIRINOX was typically reserved for patients with a good performance status (ECOG 0–1), whereas GEM-ABX was preferred in patients with a slightly lower functional status, in line with NCCN Guidelines for Pancreatic Adenocarcinoma [18,19].

SBRT was administered using a simultaneous integrated boost (SIB) strategy with three predefined dose-escalation levels—45 Gy in 5 fractions (9 patients), 50 Gy in 5 fractions (18 patients), and 55 Gy in 5 fractions (9 patients). In addition, a prophylactic PTV33 was treated with 33 Gy in 5 fractions to account for potential microscopic disease extension. Dose prescription followed institutional SBRT standards, aiming for a target coverage of D98% ≥ 98% for the CTV (acceptable threshold ≥95%), while limiting the maximum dose to < 107%. Plans were normalized to the isodose line encompassing the PTV, which was typically between 80% and 85%. The mean prescribed dose in the entire cohort was 49.9 Gy (range: 45–55 Gy). All patients completed the scheduled treatment and no acute or late severe (≥grade 3) gastrointestinal toxicity was observed. Patients were followed every 3 months during the first two years, and every six months, thereafter. As previously described [1], the follow-up included physical examination, general blood tests, and CT/MRI every 3 months for the first year, as well as every 6 months thereafter or when there was clinical suspicion of relapse.

The current study focused exclusively on the prognostic value of pre-radiotherapy inflammatory markers, namely the platelet-to-lymphocyte ratio (PLR) and neutrophil-to-lymphocyte ratio (NLR), measured immediately before SBRT initiation. The study was approved by the Institutional Review Board of the University Hospital Dr. Negrín (Las Palmas) and registered under EudraCT Number: 2019–001715-23. Written informed consent was obtained from all patients prior to treatment initiation, in accordance with international ethical standards [20].

### 2.2. Inflammatory Markers

Inflammatory markers were studied in all patients by complete blood count tests before SBRT initiation and data were recorded in the clinical chart. Systemic inflammatory markers were calculated as recommended [21]. The platelet-to-lymphocyte ratio (PLR) was obtained by dividing the platelet count by the lymphocyte count. The neutrophil-to-lymphocyte ratio (NLR) was obtained by dividing the neutrophil count by the lymphocyte count. As recommended [22,23,24], for both PLR and NLR, patients were stratified into high vs. low groups according to the median value of the distribution. For both PLR and NLR, patients were stratified into high and low groups based on the median value of the cohort at the pre-SBRT time point [8,10,14].

### 2.3. Study Endpoints

The primary endpoint of the study was to assess the role of pre-treatment PLR/NLR in predicting freedom from local progression as the first site of failure (FFLP-FF) in the context of dose-escalated SBRT. FFLP-FF is defined as the time from diagnosis to radiologic confirmation of local tumor progression, only when this was the initial site of treatment failure, in the absence of prior or synchronous distant metastasis. Patients who developed metastatic progression or died before local progression were censored at the time of the competing event. Secondary endpoints were the role of pre-treatment PLR/NLR in predicting cause-specific survival (CSS) and overall survival (OS).

### 2.4. Statistical Analysis

Categorical variables were compared using the Chi-square test or Fisher’s exact test, as appropriate. Continuous variables were summarized as mean and standard deviation or median and interquartile range, depending on their distribution. Survival outcomes, including freedom from local progression as first failure (FFLP-FF), cancer-specific survival (CSS), and overall survival (OS), were estimated using the Kaplan–Meier method. Differences between groups (e.g., high vs. low NLR/PLR) were assessed with the log-rank test. Associations between inflammatory markers and clinical outcomes were explored in univariate analyses. Multivariate analyses were not considered, given the limited number of cases in the present study. All statistical analyses were performed using IBM SPSS Statistics, version 26.0 (IBM Corp., Armonk, NY, USA). A two-sided *p*-value < 0.05 was considered statistically significant.

Follow-up was reported as the mean observed time from treatment initiation to last contact. Although the reverse Kaplan–Meier method is generally recommended to estimate the median follow-up in survival analyses [25], in our cohort, only one patient (3%) was censored, which precluded obtaining a reliable estimate. Therefore, we considered the mean follow-up and range as the most informative descriptive measures in this dataset. The closing follow-up date for survival analyses was 18 June 2025.

### 2.5. Ethical Considerations

This study was conducted in accordance with the principles of the Declaration of Helsinki and was approved by the Institutional Review Board of the University Hospital of Gran Canaria Dr. Negrín (Las Palmas, Spain) [20].

## 3. Results

### 3.1. Oncologic Outcomes of the Cohort

The median follow-up time could not be estimated due to the low number of censored cases; therefore, the mean follow-up was reported, which was 24.1 months (range, 6–71). The median overall survival (OS) was 19.0 months (95% CI: 15.8–22.2), while the mean OS was 24.1 months (95% CI: 19.1–29.1). At 2 years, the estimated cancer-specific survival (CSS) rate was 44% (SE: 9.5; median CSS: 23.0 months; 95% CI: 15.2–30.8), while the freedom from local progression as first failure (FFLP-FF) rate was 80.2% (95% CI: 72.7–87.7). At the time of analysis, only one patient (3.0%) remained alive.

### 3.2. Inflammatory Marker Distribution

Pre-SBRT values of systemic inflammatory markers were analyzed. The median PLR was 88.68 (range: 0.81–1670.00), and the mean value was 154.35 (SD: 292.49). For NLR, the median was 2.18 (range: 0.53–64.00), with a mean value of 4.08 (SD: 11.18). Eight patients had a PLR and NLR over the median (High PLR and High NLR group).

### 3.3. Association with Freedom from Local Progression as First Failure (FFLP-FF)

**Figure 1:**
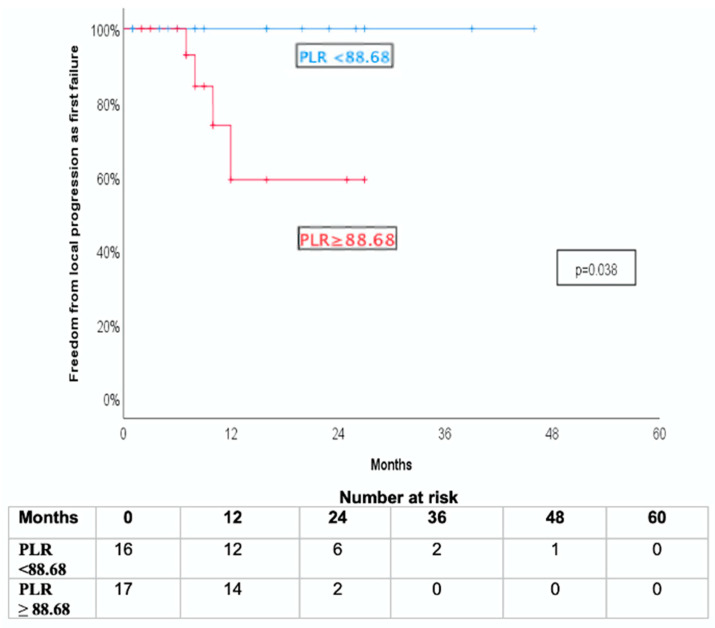
Kaplan-Meier curve for freedom from local progression as first failure (FFLP-FF) stratified by pre-SBRT platelet-to-lymphocyte ratio (PLR).

A significant association was observed between systemic inflammatory markers and local tumor control. Patients with pre-SBRT PLR values below the median (88.68) exhibited significantly improved freedom from local progression as the first site of failure (FFLP-FF) compared to those with high PLR values (log-rank *p* = 0.038; Figure 1).

**Figure 2:**
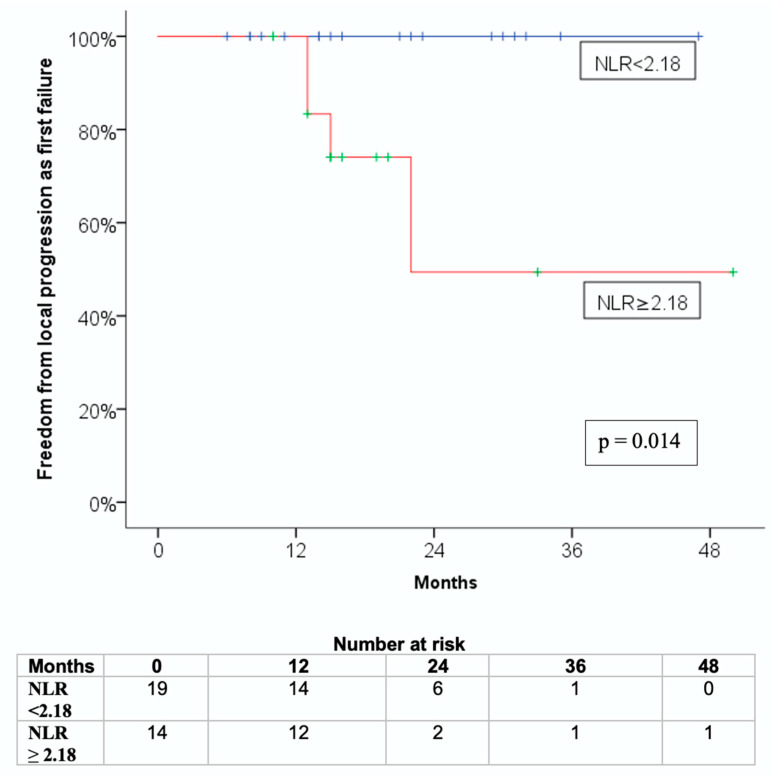
Kaplan-Meier curve for freedom from local progression as first failure (FFLP-FF) stratified by pre-SBRT neutrophil-to-lymphocyte ratio (NLR).

Similarly, patients with pre-SBRT NLR values below the median showed significantly better FFLP-FF rates than those in the high NLR group (log-rank *p* = 0.014; Figure 2).

**Figure 3:**
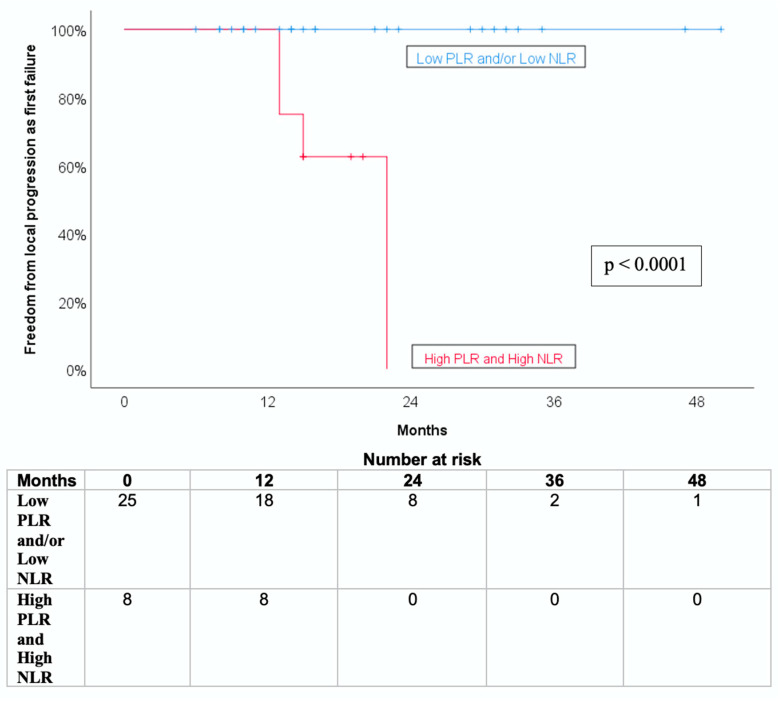
Kaplan-Meier curve for freedom from local progression as first failure (FFLP-FF) stratified by pre-SBRT platelet-to-lymphocyte ratio (PLR) and neutrophil-to-lymphocyte ratio(NLR).

A composite variable was created to identify patients with both elevated pre-SBRT PLR and NLR values (above the median). This subgroup (n = 8) exhibited a significantly poorer freedom from local progression as first failure (FFLP-FF) compared to the rest of the cohort (n = 25), with a 50% event rate versus 0%, respectively (log-rank *p* <0.0001; Figure 3).

### 3.4. Association with Overall Survival and Cancer-Specific Survival

**Figure 4:**
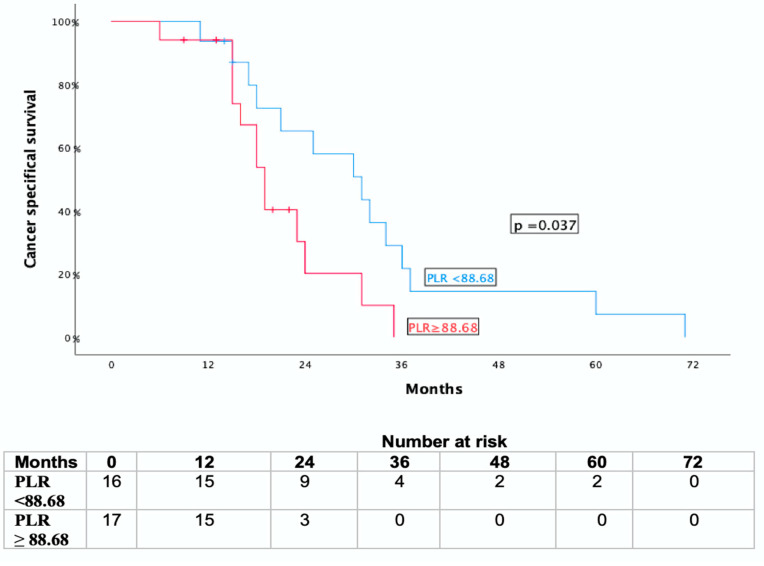
Kaplan-Meier curve for cancer-specific survival (CSS) and overall survival (OS) stratified by pre-SBRT platelet-to -lymphocyte ratio (PLR).

**Figure 5:**
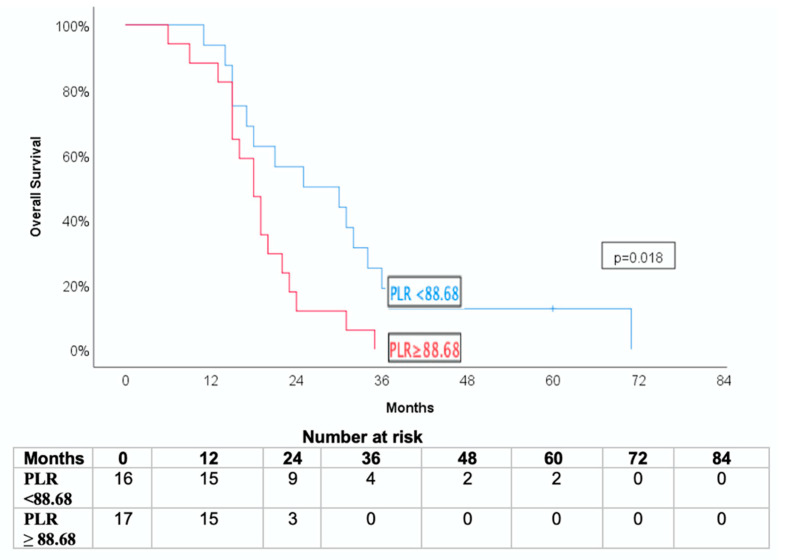
Kaplan-Meier curve for overall survival (OS) stratified by pre-SBRT platelet-to-lymphocyte ratio (PLR).

A statistically significant association was observed between systemic inflammatory markers and survival outcomes. Patients with high PLR values prior to SBRT (≥ median, 88.68) experienced a shorter CSS and OS than those with low PLR values (*p* = 0.037 and *p* = 0.018, respectively) (Figure 4 and Figure 5).

The 2-year estimates for overall survival (OS), cancer-specific survival (CSS), and freedom from local progression as first failure (FFLP-FF) according to pre-treatment systemic inflammatory markers are summarized in Table 2.

## 4. Discussion

This study demonstrates, for the first time, that systemic inflammatory markers, specifically the platelet-to-lymphocyte ratio (PLR) and neutrophil-to-lymphocyte ratio (NLR), measured before escalated high-dose stereotactic body radiotherapy (SBRT), are significantly associated with freedom from local relapse as first failure. PLR is also strongly related to survival outcomes in patients with borderline resectable (BRPC) and locally advanced pancreatic cancer (LAPC) undergoing escalated-high dose SBRT. Elevated pre-SBRT PLR was associated with poorer overall survival (OS) and cancer-specific survival (CSS), as well as a higher risk of local progression as the first site of failure (FFLP-FF). Similarly, elevated pre-SBRT NLR correlated with worse FFLP-FF rates. Furthermore, the combination of a high PLR and a high NLR prior to SBRT showed an even stronger association with decreased freedom from local progression, highlighting the additive prognostic value of these markers when considered together.

Our findings are consistent with several meta-analyses and large cohort studies that have established the prognostic significance of NLR and PLR in pancreatic cancer [8,10,14,26,27]. For instance, Yang et al. [10] and Ganbarli et al. [8] demonstrated that elevated NLR and PLR are associated with worse survival in both resectable and advanced disease. Zhou et al. [14] and Ignatavicius et al. [26] further confirmed the prognostic value of PLR, particularly in resected pancreatic cancer. Notably, García-Herrera et al. [15] recently showed that combining NLR with CA 19-9 enhances prognostic accuracy, supporting the additive value of multiple biomarkers. In our cohort, patients with a low baseline PLR and NLR demonstrated significantly improved local control compared to those with elevated values, suggesting that this subgroup may experience the most clinically meaningful benefit from escalated-dose SBRT. In contrast, patients with persistently high systemic inflammation had poorer outcomes, highlighting the need for novel approaches in this population. A potential avenue worth exploring is the combination of SBRT with immunotherapy, as radiation may enhance antigen release and immune activation, potentially counterbalancing the tumor-promoting effects of systemic inflammation. Stratification by inflammatory indices could therefore guide future trials investigating SBRT–immunotherapy combinations in pancreatic cancer.

In the context of SBRT, our results align with previous reports indicating that systemic inflammation-based markers can predict outcomes in patients receiving advanced radiotherapy modalities [4,6]. Alagappan et al. [28] specifically identified NLR as a predictor of survival in patients treated with SBRT, while Shouman et al. [6] and Petrelli et al. [4] highlighted the importance of patient selection and prognostic stratification in this setting. The clinical utility of these biomarkers lies in their accessibility, cost-effectiveness, and reproducibility, making them attractive tools for routine prognostic assessment [29].

Similarly, a higher NLR has been correlated with worse survival and increased metastatic risk, reflecting the detrimental impact of systemic inflammation on tumor biology and immune surveillance [8,12,23,24]. While most previous studies have focused predominantly on OS or progression-free survival (PFS), fewer have explored the relationship between these markers and local tumor control.

Our study contributes with novel evidence supporting the role of inflammatory markers in predicting local progression after escalated high-dose SBRT, which is a crucial clinical endpoint given the increasing emphasis on durable local control in BRPC and LAPC management [2,3,5,20,25].

Lee et al. [30], Asari et al. [31], and Hoshimoto et al. [32] confirmed the independent prognostic significance of both NLR and PLR in pancreatic cancer patients undergoing chemoradiotherapy or surgery. Moreover, Li et al. [24] and Xiao et al. [33] provided further evidence that these markers are robust predictors of survival across different treatment modalities and patient populations.

Importantly, the additive prognostic effect of a combined high PLR and NLR observed in our cohort is consistent with the hypothesis that multiple inflammatory pathways collectively influence tumor behavior and patient outcomes [15,23]. García-Herrera et al. [15] and Li et al. [23] have shown that integrating several inflammatory markers or combining them with established tumor markers such as CA 19-9 can enhance prognostic accuracy and better stratify patients at risk of poor outcomes.

Furthermore, our findings align with the growing recognition of the importance of local tumor control in the management of BRPC and LAPC, particularly in the era of dose-escalated SBRT and intensified neoadjuvant strategies [5,34,35]. Several recent studies have highlighted that improved local control translates into better quality of life and may even impact long-term survival, underscoring the clinical relevance of identifying patients at higher risk of local progression [35,36].

Overall, our study adds to the expanding body of literature supporting the use of systemic inflammatory markers as accessible, cost-effective, and reproducible prognostic tools in pancreatic cancer, and provides new insights into their role in predicting local tumor control in the context of modern multimodal therapy.

The routine measurement of PLR and NLR, derived from standard complete blood counts, offers a practical and cost-effective approach to augment existing clinical risk stratification models. The prognostic value of systemic inflammation has been widely recognized in oncology [37]. Multiple meta-analyses and cohort studies, including those in resected pancreatic cancer, have confirmed that elevated pre-treatment PLR and NLR are robust predictors of poor overall survival and progression-free survival, regardless of disease stage or treatment modality [25,38,39,40]. Patients exhibiting elevated pre-SBRT PLR and/or NLR may be at higher risk for local progression and poor survival, thus warranting intensified monitoring or therapeutic modification. The identification of patients with a combined high PLR and NLR as a particularly high-risk subgroup could guide individualized treatment decisions, including prioritization for surgical conversion following neoadjuvant therapy [38] or enrollment in clinical trials [39] assessing novel systemic or local approaches [40,41]. Incorporating these biomarkers into multidisciplinary tumor board discussions may enhance clinical decision-making and improve personalized care for BRPC and LAPC patients.

Furthermore, the integration of PLR and NLR into composite prognostic models, alongside established clinical and molecular factors, may enhance the precision of risk stratification and facilitate the development of personalized treatment algorithms [15,42]. The biological rationale for the prognostic value of these markers is supported by evidence linking systemic inflammation to tumor progression, immune evasion, and metastatic potential [43]. As the therapeutic landscape for BRPC and LAPC continues to evolve, the adoption of accessible and reproducible biomarkers such as PLR and NLR will be essential for optimizing patient outcomes and advancing precision oncology [41,44].

We consider that our prospective study has some strengths, as it is performed on a homogeneous cohort of patients with borderline resectable (BRPC) and locally advanced pancreatic cancer (LAPC), all of them treated under a standardized neoadjuvant protocol that included dose-escalated stereotactic body radiotherapy (SBRT). This uniformity in patient selection and treatment enhances the internal validity of our findings and allows for a robust evaluation of the prognostic value of inflammatory biomarkers. A further strength is the focus on clinically meaningful endpoints, such as freedom from local progression, in addition to traditional survival outcomes. This approach reflects real-world clinical priorities and addresses the growing emphasis on durable local control and conversion to surgery in the management of BRPC and LAPC. The multidisciplinary management of all patients, including standardized staging, treatment planning, and follow-up, minimized potential sources of bias and ensured consistency across the cohort.

Finally, the use of simple, inexpensive, and widely available hematological parameters—such as the neutrophil-to-lymphocyte ratio (NLR) and platelet-to-lymphocyte ratio (PLR)—underscores the immediate clinical applicability of our findings. These biomarkers can be readily integrated into routine clinical practice and multidisciplinary tumor board discussions, facilitating risk stratification and personalized treatment planning for patients with pancreatic cancer [34,35].

In our opinion, several limitations of our study merit consideration. The relatively small sample size limits the generalizability of our findings and increases susceptibility to selection bias. Although significant associations were observed, particularly for PLR and the combined PLR–NLR variables, the exploratory nature of the analysis warrants cautious interpretation. The absence of standardized cut-off values for PLR and NLR complicates direct comparisons with other studies and may impact reproducibility. The use of median values as cut-offs for immune markers, while providing robustness to the statistical analysis, may not correspond to biologically optimal thresholds. The study did not evaluate other inflammatory or immune biomarkers, nor did it explore longitudinal changes in PLR and NLR beyond the defined pre-treatment timepoints. Moreover, although we used Kaplan–Meier survival estimates and log-rank tests, which are standard and widely adopted in oncological studies, alternative approaches such as competing risks analysis could provide additional insights, particularly in differentiating local versus distant progression as first failure events. Finally, the low proportion of censored patients limited the estimation of median follow-up using the reverse Kaplan–Meier method; therefore, only mean follow-up and range could be reliably reported.

Importantly, while PLR and NLR are currently regarded primarily as prognostic biomarkers, their potential as modifiable parameters deserve further investigation. These indices reflect the interplay between systemic inflammation and immune competence, both of which may be influenced by anticancer treatments such as chemotherapy, radiotherapy, or emerging immunotherapy combinations. In addition, supportive strategies—including nutritional optimization or anti-inflammatory interventions—could contribute to modulating systemic inflammation, although robust evidence in pancreatic cancer is still lacking. Future studies should clarify whether improving systemic inflammatory status may translate into better oncologic outcomes and determine whether PLR and NLR could ultimately evolve from prognostic to predictive or even therapeutic biomarkers. Future studies with larger patient cohorts should consider such methodologies to refine outcome evaluation.

Therefore, we consider that prospective multicenter studies with larger cohorts are necessary to validate these findings and to establish standardized, clinically relevant cut-off points for PLR and NLR in pancreatic cancer. Integrating these inflammatory markers into composite prognostic models and clinical algorithms could enhance predictive accuracy and guide treatment personalization. Investigating the biological mechanisms linking systemic inflammation to tumor progression may identify novel therapeutic targets. Finally, international collaboration to harmonize biomarker reporting standards will facilitate meta-analyses and clinical guideline development. Randomized clinical trials assessing biomarker-guided treatment modifications could provide high-level evidence to optimize outcomes in this challenging disease [8,10,14,32,38].

## 5. Conclusions

This study demonstrates that both the platelet-to-lymphocyte ratio (PLR) and neutrophil-to-lymphocyte ratio (NLR), when measured prior to stereotactic body radiotherapy (SBRT), are useful prognostic markers for freedom from local progression as first failure in patients with borderline resectable and locally advanced pancreatic cancer treated with neoadjuvant SBRT. These simple and inexpensive blood-based markers can help clinicians identify patients at greater risk of early local progression and poorer outcomes.

Importantly, we observed that patients with simultaneous elevation of both PLR and NLR before SBRT faced a markedly higher risk of local failure as the first site of progression. This finding highlights the added value of combining multiple inflammatory indices to refine risk stratification and guide more personalized treatment decisions. Further research is needed to validate optimal cut-off values for PLR and NLR in larger, prospective cohorts in order to assess their changes during the course of neoadjuvant therapy, as well as to better understand the biological links between systemic inflammation and tumor behavior.

## Figures and Tables

**Table 1:** Baseline clinical and demographic characteristics.

Characteristic	Number of Patients (%)
Sex	
Male	14 (42.4%)
Female	19 (57.6%)
Histology	
Adenocarcinoma	33 (100%)
Stage T	
T2	6 (18.2%)
T3	15 (45.5%)
T4	12 (36.3%)
Stage N	
N0	25 (75.8%)
N1	6 (18.1%)
N2	2 (6.1%)
Stage M	
M0	33 (100%)
Tumor classification	
Borderline (BRPC)	13 (39.4%)
Locally advanced pancreatic cancer (LAPC)	20 (60.6%)

**Table 2:** Two-year survival outcomes stratified by pre-treatment systemic inflammatory markers (PLR, NLR, and combined PLR/NLR).

	Freedom from Local Progression as First Failure (FFLP-FF 2 Years)	Cancer-Specific Survival (CSS) (2 Years)	Overall Survival (OS) 2 Years
PLR			
Low (<88.68)	77.4% ± 9.8%	63.2% ± 12.6%	11.8% ± 7.8%
High (≥88.68)	45.2% ± 12.7%	40.3% ± 12.7%	47.1% ± 12.1%
	*p* = 0.038	*p* = 0.037	*p* = 0.018
NLR			
Low (<2.18)	71.8% ± 13.1%	45.1% ± 12.5%	42.1% ± 11.3%
High (≥2.18)	11.1% ± 10.5%	36.9% ± 13.8%	21.4% ± 11.0%
	*p* = 0.014	*p* = 0.886	*p* = 0.597
PLR/NLR			
Low	100% ± 0.0%	50.8% ± 10.7%	44% ± 9.9%
High	50.0% ± 17.1%	28.6% ± 17.1%	0.0% ± 0.0%
	*p* < 0.001	*p* = 0.224	*p* = 0.041

Abbreviations—OS: overall survival; CSS: cancer-specific survival; FFLP-FF: freedom from local progression as first failure; PLR: platelet-to-lymphocyte ratio; NLR: neutrophil-to-lymphocyte ratio; SBRT: stereotactic body radiotherapy. Note: Values represent 2-year survival estimates ± standard error. “Low” and “High” groups were defined using the median values of each biomarker. The combined PLR/NLR “High” group includes patients with both PLR and NLR above the respective medians.

## Data Availability

The data presented in this study are available upon request from the corresponding author due to ethical reasons.
